# Supplementary material for: Human fetal cartilage-derived chondrocytes and chondroprogenitors display a greater commitment to chondrogenesis than adult cartilage resident cells
Source: PLoS One. 2023 Apr 27;18(4):e0285106. doi: 10.1371/journal.pone.0285106 (PMC10138236; doi:10.1371/journal.pone.0285106)
Supplement: S1 Table — MSC: Mesenchymal stem cell, CD: Cluster of differentiation, FITC: Fluorescein isothiocyanate, PE: Phycoerythrin, APC: Allophycocyanin, BB515: Horizon brilliant blue 515, BV421: Brilliant violet 421 and V500: Violet 500. (DOCX) [file pone.0285106.s001.docx]

S1 Table: List of antibodies used for characterization of chondroprogenitors by flow cytometric analysis. MSC: mesenchymal stem cell, CD: cluster of differentiation, FITC: fluorescein isothiocyanate, PE: phycoerythrin, APC: allophycocyanin, BB515: Horizon brilliant blue 515, BV421: Brilliant violet 421 and V500: Violet 500.

| **Groups** | **Surface Markers** | | **Fluorochrome**  **Conjugate** | **Catalogue number** | **Source** |
| --- | --- | --- | --- | --- | --- |
| **Group I:**  Positive MSC markers | CD105: Endoglin glycoprotein | | FITC | 561443 | BD Bioscience |
|  | CD73: Ecto-5’- nucleotidase | | PE | 550257 | BD Bioscience |
|  | CD90: Thymus cell antigen 1 | | PE | 561970 | BD Bioscience |
|  | CD106 | | APC | 551147 | BD Bioscience |
| **Group II:**  Negative MSC markers | CD34 | Hematopoietic stem cell markers | PE | 348057 | BD Bioscience |
|  | CD45 |  | FITC | 347463 | BD Bioscience |
|  | CD14: Monocyte/macrophage marker | | FITC | 555397 | BD Bioscience |
| **Group III:**  Integrin markers | CD29: Integrin beta-1 (Iβ1) | | APC | 559883 | BD Bioscience |
|  | CD49e: Integrin alpha 5; (Iα5) Fibronectin receptor | | PE | 555617 | BD Bioscience |
|  | CD49b: Integrin alpha 2; (Iα2) | | FITC | MACS 130/100337 | Miltenyl Biotec |
| **Group IV:**  Potential markers of enhanced chondrogenesis | CD146: Melanoma cell adhesion molecule | | PE | 550315 | BD Bioscience |
|  | CD166: Activated leucocyte adhesion molecule | | BB515 | 565461 | BD Bioscience |
|  | Podoplanin: Type I integral membrane glycoprotein | | BV421 | 566456 | BD Bioscience |
| **Group IV:**  Immunogenic markers | HLA-ABC: Human Leukocyte Class I | | PE | 560964 | BD Bioscience |
|  | HLA-DR: Human Leucocyte Class II | | V500 | 561225 | BD Bioscience |
|  | CD80: HLA-II costimulatory marker | | BB515 | 565009 | BD Bioscience |
|  | CD86: HLA-II costimulatory marker | | BV421 | 562433 | BD Bioscience |
