# Supplementary material for: Human fetal cartilage-derived chondrocytes and chondroprogenitors display a greater commitment to chondrogenesis than adult cartilage resident cells
Source: PLoS One. 2023 Apr 27;18(4):e0285106. doi: 10.1371/journal.pone.0285106 (PMC10138236; doi:10.1371/journal.pone.0285106)
Supplement: S2 Table — SOX9: Sex determining region Y-box 9, ACAN: Aggrecan, COL2A1: Collagen type 2 alpha 1 chain, COL1A1: Collagen type 1 alpha 1 chain, COL10A1: Collagen type 10 alpha 1 chain, RUNX2: Runt related transcription factor-2 and GAPDH: Glyceraldehyde 3-phosphate dehydrogenase. (DOCX) [file pone.0285106.s002.docx]

S2 Table: Sequence of the primers used for RT-PCR. SOX9: sex determining region Y-box 9, ACAN: Aggrecan, COL2A1: Collagen type 2 alpha 1 chain, COL1A1: Collagen type 1 alpha 1 chain, COL10A1: Collagen type 10 alpha 1 chain, RUNX2: Runt related transcription factor-2 and GAPDH: glyceraldehyde 3-phosphate dehydrogenase

| **Gene of Interest** | **Primers (5’-3’)** | | **Accession number (reference link)** | **Product size**  **(bp)** |
| --- | --- | --- | --- | --- |
|  | **Forward primer** | **Reverse primer** |  |  |
| **SOX-9** | GACTTCCGCGACGTGGAC | GTTGGGCGGCAGGTACTG | NM_000346.4  <https://www.ncbi.nlm.nih.gov/nucleotide/1519242934> | 99 |
| **ACAN, transcript variant 1** | TCGAGGACAGCGAGGCC | TCGAGGGTGTAGCGTGTAGAGA | NM_001135.4  <https://www.ncbi.nlm.nih.gov/nucleotide/1890265422> | 85 |
| **COL2A1, transcript variant 2** | CCTGAGTGGAAGAGTGGAGAC | TTGCTGCTCCACCAGTTCTT | NM_033150.3  <https://www.ncbi.nlm.nih.gov/nucleotide/1674985896> | 149 |
| **COL1A1** | TCTGCGACAACGGCAAGGTG | GACGCCGGTGGTTTCTTGGT | NM_000088.4  <https://www.ncbi.nlm.nih.gov/nucleotide/1777425449> | 146 |
| **RUNX2,**  **transcript variant 2** | CCTAAATCACTGAGGCGGTC | CAGTAGATGGACCTCGGGAA | NM_001015051.4  <https://www.ncbi.nlm.nih.gov/nucleotide/1890358904> | 91 |
| **GAPDH**  **transcript variant 7** | TCAGCAATGCCTCCTGCAC | TCTGGGTGGCAGTGATGGC | NM_001357943.2  <https://www.ncbi.nlm.nih.gov/nucleotide/1676440496> | 117 |
