# Supplementary material for: Human fetal cartilage-derived chondrocytes and chondroprogenitors display a greater commitment to chondrogenesis than adult cartilage resident cells
Source: PLoS One. 2023 Apr 27;18(4):e0285106. doi: 10.1371/journal.pone.0285106 (PMC10138236; doi:10.1371/journal.pone.0285106)
Supplement: Attachment — Submitted filename: Response to Reviewers comments.docx [file pone.0285106.s005.docx]

| Authors would like to sincerely thank the reviewers for taking the time to go through the manuscript and providing a detailed review. We appreciate the opportunity to provide clarifications and modify our manuscript accordingly. | | |
| --- | --- | --- |
| S.No | Editor/Reviewer comment(s) | Author response and modification |
| 1. | Additional Editor Comments |  |
|  | This paper is essentially provisionally accepted pending very minor final revisions of the paper. | The authors thank the editor for this comment and have made the required modifications to the manuscript. |
| 2. | Reviewer #1: | |
|  | Reviewer #1:  The reviewers have addressed all comments and have some minor edits that would benefit the manuscript.   1. Figure 7: the images in the SafraninO column look like Alcian Blue and the images in the Alcian Blue column look like Safranin O. Please check and correct if necessary   2. line 110: remove the work "remarkable" . It is not needed.  3. line 415: need a reference about the Phase I clinical trial mentioned.  4. line 483: would benefit from some references | The authors thank the reviewer for the suggestions and have made the required modifications to the manuscript.  The authors have rechecked the figures and ensured that the grid is correct. Safranin O staining was performed using 1% Safranin O solution with fast green. The pellets displayed a pink to orange uptake at the areas showing the presence of proteoglycans.  In the case of Alcian blue staining, it was performed at a pH of 2.5 with a neutral red counterstain, staining areas containing sulfated and carboxylated glycosaminoglycans (blue to turquoise coloration). (Line no: 259-262)  As suggested the word remarkable has been removed. (Line No: 110)  Thank you for the suggestion, the reference has been included. (Line no: 416)  Relevant references have been included (Line No: 484) |
| 3. | Journal Requirements: |  |
|  | Journal Requirements:  Please review your reference list to ensure that it is complete and correct. If you have cited papers that have been retracted, please include the rationale for doing so in the manuscript text,or remove these references and replace them with relevant current references. Any changes to the reference list should be mentioned in the rebuttal letter that accompanies your revised manuscript. If you need to cite a retracted article, indicate the article’s retracted status in the References list and also include a citation and full reference for the retraction notice. | The authors have crosschecked the cited papers, ensuring it does not include any retracted papers.  We found that two references from the same paper were duplicated. We regret this oversight, and we have rectified the issue. |
